# 4E10-Resistant HIV-1 Isolated from Four Subjects with Rare Membrane-Proximal External Region Polymorphisms

**DOI:** 10.1371/journal.pone.0009786

**Published:** 2010-03-23

**Authors:** Kyle J. Nakamura, Johannes S. Gach, Laura Jones, Katherine Semrau, Jan Walter, Frederic Bibollet-Ruche, Julie M. Decker, Laura Heath, William D. Decker, Moses Sinkala, Chipepo Kankasa, Donald Thea, James Mullins, Louise Kuhn, Michael B. Zwick, Grace M. Aldrovandi

**Affiliations:** 1 Department of Pediatrics, Childrens Hospital of Los Angeles, Los Angeles, California, United States of America; 2 Systems Biology and Disease Program, University of Southern California Keck School of Medicine, Los Angeles, California, United States of America; 3 Department of Immunology and Microbial Science, The Scripps Research Institute, La Jolla, California, United States of America; 4 Center for International Health and Development, Boston University School of Public Health, Boston, Massachusetts, United States of America; 5 Department of Medicine, University of Alabama at Birmingham, Birmingham, Alabama, United States of America; 6 Department of Microbiology, University of Washington, Seattle, Washington, United States of America; 7 Lusaka District Health Management Team, Lusaka, Zambia; 8 University Teaching Hospital, University of Zambia, Lusaka, Zambia; 9 Department of Medicine, University of Washington, Seattle, Washington, United States of America; 10 Department of Epidemiology, Columbia University, New York, New York, United States of America; University of California San Francisco, United States of America

## Abstract

Human antibody 4E10 targets the highly conserved membrane-proximal external region (MPER) of the HIV-1 transmembrane glycoprotein, gp41, and has extraordinarily broad neutralizing activity. It is considered by many to be a prototype for vaccine development. In this study, we describe four subjects infected with viruses carrying rare MPER polymorphisms associated with resistance to 4E10 neutralization. In one case resistant virus carrying a W680G substitution was transmitted from mother to infant. We used site-directed mutagenesis to demonstrate that the W680G substitution is necessary for conferring the 4E10-resistant phenotype, but that it is not sufficient to transfer the phenotype to a 4E10-sensitive Env. Our third subject carried Envs with a W680R substitution causing variable resistance to 4E10, indicating that residues outside the MPER are required to confer the phenotype. A fourth subject possessed a F673L substitution previously associated with 4E10 resistance. For all three subjects with W680 polymorphisms, we observed additional residues in the MPER that co-varied with position 680 and preserved charged distributions across this region. Our data provide important caveats for vaccine development targeting the MPER. Naturally occurring Env variants described in our study also represent unique tools for probing the structure-function of HIV-1 envelope.

## Introduction

The membrane-proximal external region (MPER) of gp41 is an attractive target for HIV vaccine development [1], [2], [3], [4], [5]. It is highly conserved across group M HIV-1, it is not glycosylated, and its deletion renders the envelope non-fusogenic [6], [7]. The HIV-1 MPER is also the target of broadly neutralizing monoclonal antibodies including 4E10, 2F5 and Z13e1; 4E10 being the most broadly reactive neutralizing antibody to HIV-1 described to date. Human antibodies 2F5 and 4E10 were derived directly from chronically infected persons, indicating that these broad specificities can be elicited *in vivo* [8], [9], [10], [11], [12], [13]. With the recent limited performance of a major T-cell based vaccine trial [14], [15], there is renewed interest in vaccines that elicit neutralizing antibodies targeting conserved regions of Env such as the MPER [1], [2], [3], [4], [16], [17], [18].

Monoclonal antibody 4E10 neutralizes almost all Group M primary isolates in pseudotyped virus assay systems [19], [20]. The core epitope for this mAb has been crudely defined as N_671_W_672_F_673_D_674_I_675_T_676_ (HXB2 numbering), though W680 has been implicated as critical to 4E10 binding in several studies [12], [21], [22], [23]. Naturally occurring polymorphisms are extremely rare for all three crucial residues (W_672_F_673_W_680_), with substitution frequencies of 0.07%–0.43% per site (based on 2811 gp41 sequences in the 2009 LANL database). Studies of recently and chronically infected persons failed to detect naturally occurring resistance to 4E10 [19], [20] and antibodies to 4E10 epitopes are also very rare [24], [25]. Only one study has identified a subject with intrinsic 4E10 resistance that correlated with a natural polymorphism in the epitope sequence (F673L) [26], though another study identified phenotypically resistant virus without genotypic changes in the MPER regions [27]. Additionally, human passive immunotherapy trials using a combination of mAbs including 4E10 did not select for 4E10-resistant virus [28], [29], [30], and generation of resistant virus during *in vitro* passage is difficult and only partially successful [31]. For these reasons, many consider the induction of 4E10-like specificities to be an important component in the development of a protective vaccine [1], [2], [4], [5], [20], [23], [32], [33]. However, there have been limited *in vivo* trials of this mAb [28], [29], [30], [34], [35], [36], [37] and naturally occurring 4E10-like specificities appear to be rare. Therefore, little is known about potential escape paths from 4E10-like antibodies and the associated mechanisms.

In this study, we describe four subjects chronically infected with subtype C HIV-1 from whom we isolated envelopes resistant to neutralization by mAb 4E10. In all four cases, 4E10 resistance was associated with rare polymorphisms at positions 673 and 680 in the MPER. One subject transmitted a 4E10 resistant virus to her infant *in utero*. Co-variation analysis and site-directed mutagenesis showed a relatively conserved pattern of MPER substitutions associated with mutation at position 680, but also revealed that residues outside the MPER are important in conferring the 4E10-resistant phenotype. Our data show that MPER polymorphisms conferring resistance to broadly neutralizing anti-MPER antibodies occur naturally, are “fit” enough to transmit, and thus should be considered as a potential escape path if this region of Env is included in a vaccine. Our data also provide corroborating evidence for several hypotheses about Env structure/function, as well as the mechanism of 4E10 binding/neutralization.

## Materials and Methods

### Subject Data

All subjects were part of the Zambia Exclusive Breastfeeding Study (ZEBS), a clinical trial to prevent mother to child HIV-1 transmission [38]. HIV-1 infected women were enrolled antenatally and their children were followed for 24 months. All the women and infants in this analysis received a single peripartum dose of nevirapine as per the Zambian government guidelines at that time. Twenty transmitting mothers were identified, based on sample availability and quality, for genotypic and phenotypic analysis of maternal and infant *envs*. In total, out of 20 transmission-pairs analyzed, we identified four subjects (three transmitting mothers and one infant recipient) with rare polymorphisms in their MPERs associated with resistance to mAb 4E10. All other subjects harbored phenotypically 4E10-sensitive Envs with commonly observed MPER sequences.

All women signed informed consent. ZEBS was approved by Human Subjects Committees at the investigators' institutions in the US (Boston University, Columbia University, University of Alabama, Birmingham and Childrens Hospital Los Angeles) and by the University of Zambia Research Ethics Committee. Laboratory specimens were completely anonymized and unlinked.

### Cloning and Sequencing

Complete gp160 envelopes were cloned directly from plasma or cells for all study subjects as previously described by Derdeyn et al. [39], with the addition of multiple independent PCRs performed at or near limiting dilution to prevent re-sampling [40]. Cloned envelopes were sequenced bi-directionally and sequences were assembled and edited using the Sequencher software (Gene Codes), MacVector (MacVector Inc), and the Los Alamos National Laboratory (LANL) website tools (http://www.hiv.lanl.gov/content/sequence/LOCATE/locate.html).

All chromatograms were visually inspected during assembly and any with dual peaks were excluded. In the event that multiple clones were generated and sequenced from a single PCR product, nucleotide alignments were examined and if clones were identical or nearly identical only one representative sequence was retained for further analysis. All sequences were compared against the HIV-1 database using ViroBLAST [41]. Sequences were also aligned in MUSCLE v3.7 [42] and refined manually in MacClade v4.08 software (Sinauer Associates, Inc., Sunderland, MA). A maximum likelihood tree was calculated in PhyML v3.0 [43] using the online tool DIVER (http://indra.mullins.microbiol.washington.edu/cgi-bin/DIVER/diver.cgi), which implemented the evolutionary model GTR+I+G. The tree was rooted with subtype B reference sequence HXB2. Upon examination of the tree, sequences from each mother/infant pair were observed to cluster separately from every other pair, thus suggesting a lack of inter-patient or reference strain contamination.

### Cells, Inhibitors, and Other Reagents

293T cells were obtained from the American Type Culture Collection (ATCC), and TZM-bl cells were obtained from the AIDS Research and Reference Reagent Program, Division of AIDS, NIAID, NIH: (catalogue #8129) courtesy of Dr. John C. Kappes, Dr. Xiaoyun Wu and Tranzyme Inc [44], [45], [46], [47], [48]: both were maintained in Dulbecco's Modified Eagles Media (DMEM) (Fisher Scientific) supplemented with 10% Fetal Bovine Serum (Gemini Bio-products), 100 U/mL penicillin-streptomycin (Gibco), and 2mM L-Glutamine (Gibco) at 37°C with 5% CO_2_. The following plasmids were obtained through the AIDS Research and Reference Reagent Program, Division of AIDS, NIAID, NIH: *env* clone Du422 (SVPC5) (catalogue #11308, Genebank Accession# DQ411854) from Drs. D. Montefiori, F. Gao, C. Williamson, and S. Abdool Karim [49], and the backbone plasmid pSG3ΔEnv (catalogue #11051) from Drs. John C Kappes and Xiaoyun Wu [45], [50]. TZM-bl cells expressing FcγRI were kindly provided by Dr. David Montefiori and Dr. Gabriel Perez [48], [51].

The following drugs and antibodies were obtained from the AIDS Research and Reference Reagent Program, Division of AIDS, NIAID, NIH: HIV-1 gp41 Monoclonal Antibody (4E10) from Dr. Hermann Katinger (catalogue #10091); TAK-779 from Takeda Chemical Industries, Ltd. (catalogue #4983); T-20 Fusion Inhibitor from Roche (catalogue #9845). mAb 4E10 was also purchased directly from Polymun Scientific.

ELISA peptides (>95% pure by HPLC) were synthesized as described previously [9], [33] at The Scripps Research Institute (P. Dawson), recombinant gp41 (HxB2, amino acids 541–682) was purchased from Vybion (Ithaca, NY), and M41xt (gp41_JR-FL_, amino acids 535–681) was produced as a C-terminal fusion to the maltose-binding protein (MBP) in *Escherichia coli* and purified on an amylose column [52].

### Neutralization Assay

Neutralization of pseudotyped virus was measured as the reduction of luciferase activity after infection of TZM-bl cells in the presence of varying concentrations of antibody or drug, as previously described [39]. In summary, pseudotyped virus was produced in 293T cells by co-transfection of an *env* plasmid and the pSG3ΔEnv backbone. Pseudotyped virus was incubated for 1 hr with 5-fold dilutions of the test antibody/drug and then added to TZM-bl cells in a 96 well plate format in the presence of 16 µg/mL DEAE-dextran. Two days later, cells were lysed and analyzed using a Promega Luciferase kit (Promega, Madison WI) and a FLUOstar luminometer (BMG Labtech). Relative infectivity was expressed as a percentage of the drug-free control.

### Site-directed mutagenesis

The Quikchange II Multi-Site directed mutagenesis kit (Stratagene) was used to create the G680W reversion mutant and the constructs SVPC5-KGQI, SVPC5-KWKI, and SVPC5-NRQL. Mutagenesis was confirmed by full-length bi-directional sequencing.

### Statistical Analysis

Fifty percent inhibitory concentration (IC_50_) for each drug was calculated by using the data points immediately above and below 50% infectivity using the POWER function in Excel (Microsoft). IC_50_ results were averaged between at least two independent assays. Since phenotypic data were not normally distributed, the Wilcoxon rank-sum test or the Kolmogorov-Smirnov test was used to compare IC_50_ values.

### HIV-1/HIV-2 Chimera Neutralization

The HIV-1/HIV-2 Env chimeras 7312A (HIV-2 Env), 7312-C1 (HIV-2 Env with subtype B HIV-1 MPER), and 7312-C1C (HIV-2 Env with HIV-1 subtype C MPER) have been previously described [24]. Neutralization experiments were conducted using heat inactivated plasma or cell-free breast milk supernatant from subject 16M as previously described [24].

### ELISA Testing

96-well microplates (eBioscience) were incubated with test peptide or recombinant protein overnight at 4°C (100 ng/well). Plates were washed with TPBS (PBS containing 0.01% Tween 20) and blocked for 1 hr with 4% non-fat dry milk (NFDM) in TPBS. Plasma or mAb samples were serially diluted in 1% NFDM/TPBS and added to the antigen-coated wells. After 1 hr, bound antibody was probed with a peroxidase-conjugated goat anti-human IgG Fab (Sigma) diluted 1∶1000 in 1% NFDM/TPBS. Bound conjugate was detected using TMB substrate (Pierce) and the colorimetric signal measured at 450 nm.

### HIV-1 neutralization assay using FcγRI-transgenic TZM-bl cells

Pseudotyped HIV-1_JR-FL_ was added to serially diluted (1∶3) IgG variants (starting at 10 µg/ml) or human serum samples (starting at a 1∶40 dilution) and incubated at 37°C, for 1 hr. TZM-bl or TZM-blFcγRI cells were then added (1∶1 by volume) at 1×10^4^ cells/well in a final concentration of 10 µg/ml DEAE-dextran, as described previously [51]. After 48 hr incubation the cells were washed, lysed and developed using luciferase assay reagent according to the manufacturer's instructions (Promega). Luminescence was measured using an Orion microplate luminometer (Berthold Detection Systems).

### Nucleotide Sequence Accession Numbers

All *env* sequences were submitted to GenBank under accession numbers GU939049 to GU939171.

## Results

**Figure 1:**
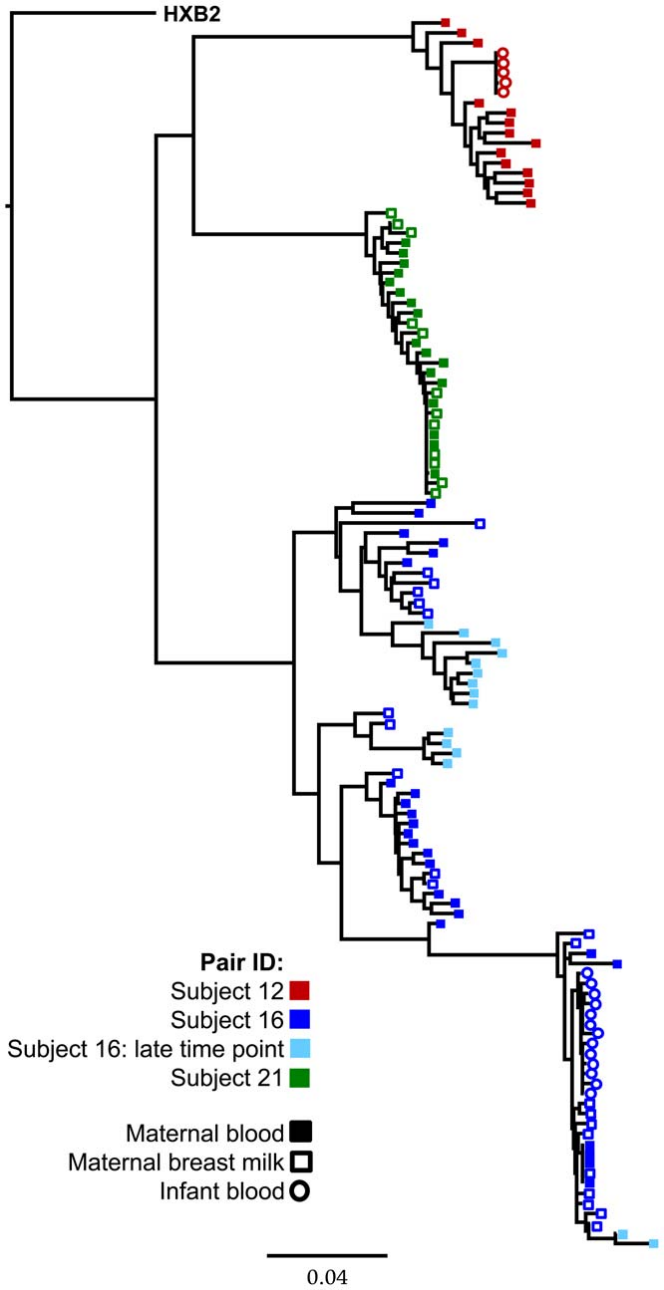
Maximum-Likelihood phylogenetic tree of all *env* sequences used in this study. HXB2 is used as an outgroup to root the tree.

While characterizing functional *env* genes from a cohort of 20 mother-infant transmission pairs, we identified clones in 3 transmitting mothers that were highly resistant to mAb 4E10. Their clinical characteristics are presented in Table 1. One of these women transmitted 4E10-resistant clones to her infant (subjects 16M & 16B), while another transmitted a sensitive clone (subject 12M). No usable specimen was available for the third subject's infant (subject 21M). A phylogenetic tree for all three mothers and two of their infants is presented as Figure 1. Each pair forms a distinct cluster, with infant sequences forming a sub-branch off the maternal tree.

**Table 1:** Clinical characteristics of study subjects.

Patient ID	Plasma Viral Load	Trans. Type*	Maternal CD4	Baby 1^st^ Positive PCR*
Subject 16	211,792	IUT	118	Birth
Subject 12	45,907	BMT	52	3 Months
Subject 21	128,120	BMT	55	4.5 Months

*Transmission type established by timing of the infant's positive DNA PCR: *In Utero* Transmission (IUT) = HIV DNA positive at birth, Breast Milk Transmission (BMT) = HIV DNA PCR positive at >1 month with negative HIV DNA PCR results prior to that timepoint.

### Subjects 16M & 16B

**Figure 2:**
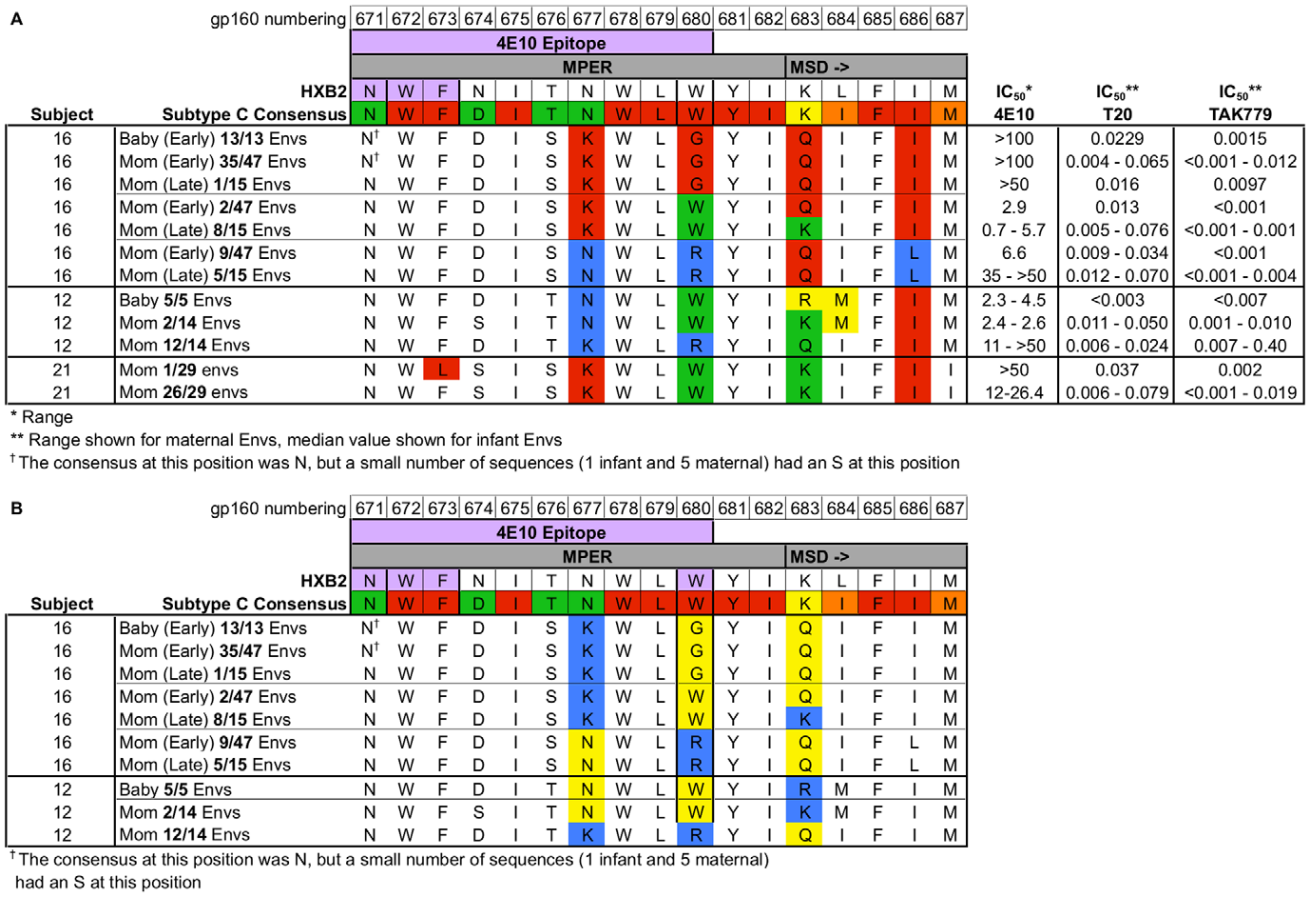
Alignment of MPER sequence variants from all subjects described in this study. Sequences are aligned against HXB2 and a subtype C consensus sequence derived from the 2007 LANL database. Numbering is based on HXB2. MSD denotes start of the Membrane Spanning Domain. Consensus residues are color-coded by degree of conservation (red >98%, orange 90–97.9%, yellow 75–89.9%, green <75%). In panel A, IC_50_ for mAb 4E10 is expressed as a range for all functional clones tested for each MPER variant. Positions 677, 680, 683, and 686 are color-coded for emphasis. In Panel B, Env protein sequences have been color-coded according to charge at positions 677, 680, and 683 (blue = basically charged residue, yellow = uncharged residue). All other residues (in white) are uncharged.

The majority of the Env clones (36/47) detected in Subject 16M possessed a glycine at position 680 instead of the usual tryptophan (W680G) and all tested clones were resistant to mAb 4E10 (IC_50_>100 µg/mL). The remaining clones had either a wild-type W (2/47) or a W680R (9/47) substitution at this position (Figure 2A). Both W680 and W680R clones were sensitive to 4E10 (IC_50_ 3.3 µg/mL and 6.6 µg/mL, respectively). One maternal clone had a W672R substitution in addition to a W680G mutation and was highly resistant to 4E10 (IC_50_>100 µg/mL)

This subject's infant was HIV DNA PCR positive at birth. Phylogenetic analysis of 13 infant *envs* revealed highly homogeneous quasi-species. All infant envelopes had a W680G substitution and were highly resistant to mAb 4E10 (IC_50_>100 µg/mL).

A maternal sample obtained 3 years after the enrollment sample was available. By this time, the distribution of MPER substitutions had changed; only 1 clone out of 15 had a W680G substitution (IC_50_>50 µg/mL), while the remaining Envs had either a W (9/15 clones) or an R (5/15 clones) at position 680. Clones with the W were susceptible (IC_50_ 0.7–5.7 µg/mL, median 3.6 µg/mL), while the R clones were more resistant (IC_50_ 35–>50 µg/mL, median 48 µg/mL) relative to both contemporaneous W clones and the early time-point R clone (Figure 2A). Unfortunately, no longitudinal infant samples were available.

In an alanine scanning mutagenesis study, Zwick et al. showed that substitution of a small, hydrophobic residue (A) at position 680 resulted in an ∼2-fold increase in susceptibility to T20 [22]. Since our W680G Envs also contain a small, hydrophobic substitution at this position, we tested the Envs from subject 16M for T20 susceptibility. The W680G Envs from this subject were ∼2-fold more susceptible to T20 than Envs with either a W or R at position 680. While this difference did not reach statistical significance (P = 0.097), we find it noteworthy that the same phenotype was observed with both *in vitro* mutagenesis of a clade B Env, and our naturally occurring clade C primary isolates.

We hypothesized that 4E10 escape mutations in this subject were the result of antibody-mediated selective pressure. To test for evidence of MPER targeted antibodies, we utilized an HIV-2/HIV-1 chimera system. HIV-2 exhibits little to no cross-neutralization with HIV-1, and mAb 4E10 does not neutralize HIV-2. This lack of cross-reactivity was exploited by grafting the subtype B and subtype C HIV-1 MPER consensus sequences into HIV-2 Envs, rendering them sensitive to neutralization by 4E10 [24]. Plasma and breast milk supernatant from this subject were tested against the chimeras; however, neutralization activity was detected against neither the subtype B (7312A-C1) nor subtype C (7312A-C1C) chimera, despite efficient neutralization of both by the 4E10 mAb control (data not shown).

**Figure 3:**
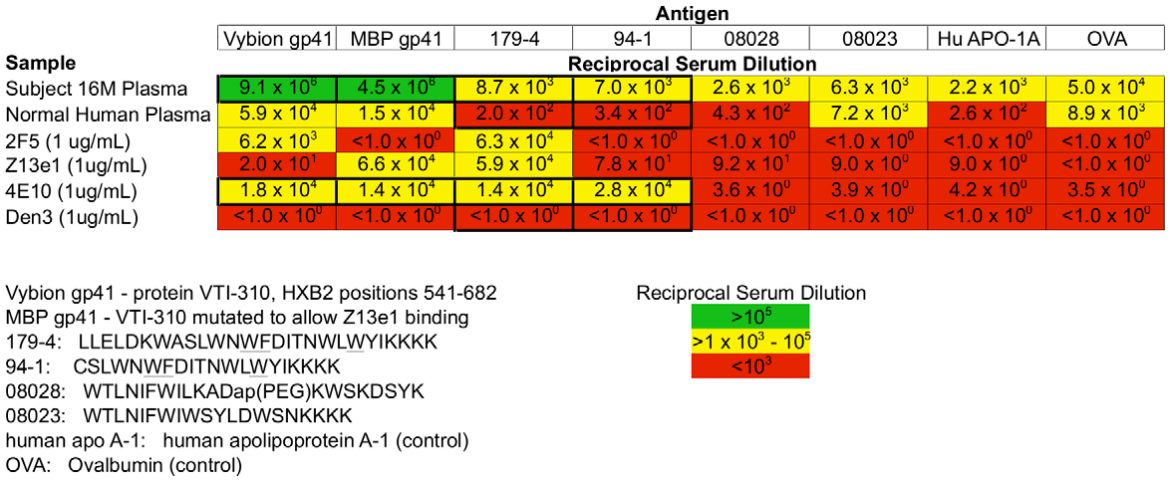
Binding ELISA results for subject 16Ms plasma. Binding ELISA testing subject 16Ms plasma against normal human plasma and four monoclonal antibodies for binding to two gp41 constructs, one full-length MPER peptide, and two control peptides.

In order to further assess the plasma from subject 16M for 4E10-like activity, we performed an ELISA against two different recombinant gp41 constructs, as well as a gp41 MPER peptide (179-4). We found that plasma from 16M bound extremely well to recombinant gp41 (>100-fold better than any of the mAb controls). Her plasma bound MPER peptide 179-4 45-fold better then the normal human plasma control. However, we also observed relatively high binding to irrelevant control proteins (hen egg white ovalbumin and human apolipoprotein A1), so these data should be interpreted with some caution. When compared to the mAb 4E10 positive control, plasma from 16M bound ∼2-fold less strongly to peptide 179-4 (Figure 3).

Perez et al. have reported that expression of FcγRI on TZM-bl cells strongly enhances neutralization of pseudotyped virus by MPER-specific broadly neutralizing mAbs 4E10 and 2F5, and that such enhancing activity is not observed with the gp120 targeted mAb 2G12, or most HIV+ plasmas (the exception was a plasma shown to contain anti-MPER activity) [51]. We tested the 16M plasma for neutralization enhancement in this assay system, finding none, despite observing a >35-fold reduction in IC_50_ for mAb 4E10 (data not shown).

### Subject 12M

The majority of the maternal *envs* (12/14) in this subject had a W680R substitution, with a minor fraction (2/14) possessing the wild-type W680. The W680R Envs had a wide range of susceptibility to 4E10 (IC_50_ 11 to >50 µg/mL), suggesting that position 680 was not the sole determinant of 4E10 sensitivity for this subject. Examination of the entire MPER did not reveal any additional mutations associated with 4E10 sensitivity in the W680R Envs. The W680 Envs were sensitive to 4E10 (IC_50_ 2.4–2.6 µg/mL). Subject 12M's infant was infected by breast milk between 2 and 3 months of age. Phylogenetic examination revealed a highly homogeneous infant *env* population. All infant Envs (5/5) were sensitive to 4E10 (IC_50_ 2.3–4.5 µg/mL), and were wild type at position 680 (W) (Figure 2A).

### Subject 21M

Subject 21M harbored predominantly wild type, 4E10-sensitive virus (28/29 clones, IC_50_ 12.0–26.4 µg/mL). A single 4E10-resistant Env (IC_50_>50 µg/mL) was isolated from this subject (Figure 2A). The 4E10-resistant clone had the F673L substitution previously described by Gray et al [26]. Some of the previously described F673L Envs also had substitutions in the lentiviral lytic peptide – 2 (LLP-2) domain of the gp41 cytoplasmic tail. These substitutions (E783A, T784I, G789V, T792L) were not present in any of the *envs* from our subject. No infant sample was available.

### MPER Sequence Analysis

Analysis of the MPER sequences from subjects 16M & 16B revealed that three residues (positions 677, 683, and 686) varied based on the identity of position 680 (Figure 2A). Some of these substitutions were themselves extremely rare (<0.01%), suggesting that they may be important compensatory mutations. The wild-type W at position 680 was associated with a K at positions 677 and 683, and an I at position 686. The W680G mutation was associated with a K at position 677, a Q at position 683, and an I at position 686. The W680R mutation was associated with an N at position 677, a Q at position 683, and an L at position 686. The presence of an uncharged amino acid at position 680 (either W or G) was associated with a charged residue (K) at position 677 and either an uncharged Q or a charged K at position 683 (for 680G or 680W respectively) (Figure 2B). The presence of a charged amino acid (R) at position 680 was associated with uncharged residues at both positions 677 (N) and 683 (Q). This co-variation was strictly conserved at both time-points sampled, and across both major branches of the maternal tree.

Envelopes from subjects 12M & 12B also had 3 residues (677, 683, and 684) that co-varied with position 680 (Figure 2A). In this subject, the wild type (680W) was associated with an uncharged residue at position 677 (N) and a charged residue at position 683 (K in maternal and R in infant Envs) (Figure 2B). The W680R mutation was associated with a charged residue at position 677 (K) and an uncharged residue at position 683 (Q). The W680 Envs from this subject also had a very rare I684L substitution in the membrane-spanning domain (present in both maternal and infant W680 Envs).

We found it particularly noteworthy that positions 677 and 683 co-varied based on the identity of position 680 in both of these subject-pairs. While the specific substitution patterns were different, we did observe similar patterns of charge conservation in both subject-pairs: the presence of a charged residue at position 680 (R) resulted in a change in the charge distribution at positions 677 and 683. We also noted that all Envs maintained either 1 or 2 basic charges across this region of gp41 (677–686). An alignment of MPER protein sequences for all subjects is presented in Figure 2A. An alignment of subject pairs 12 and 16 is presented in Figure 2B as a simplified charge map (with basic charges shown in blue). It is notable that the basic charge at position 683 is >99.9% conserved in subtype C (either as K or R), and has been proposed to serve as a membrane anchor/MSD stop signal [53] yet we observed frequent substitutions at this position linked to the presence of a G or R at position 680.

Since our data on 4E10 susceptibility were so striking, we sought to determine whether MPER substitutions were causing a global decrease in sensitivity to entry inhibitors by testing our Envs for sensitivity to the gp41 targeted fusion inhibitor T20 and the gp120 targeted inhibitor TAK-779 (Figure 2A). We did not find any major qualitative differences in sensitivity to these inhibitors based on MPER sequence. Furthermore, we found that our infant Envs fell within or very close to the range of IC_50_s present in maternal Envs. This suggests that the MPER substitutions described have not resulted in broad changes to neutralization sensitivity.

### Site-Directed Mutagenesis

To confirm that the W680G substitution in subject-pair 16 was essential for conferring the 4E10-resistant phenotype, we conducted site-directed mutagenesis to revert an infant envelope to the wild type (G680W). This single mutation increased sensitivity to 4E10 by >30-fold (IC_50_ changed from >100 µg/mL to 3 µg/mL) (data not shown).

**Figure 4:**
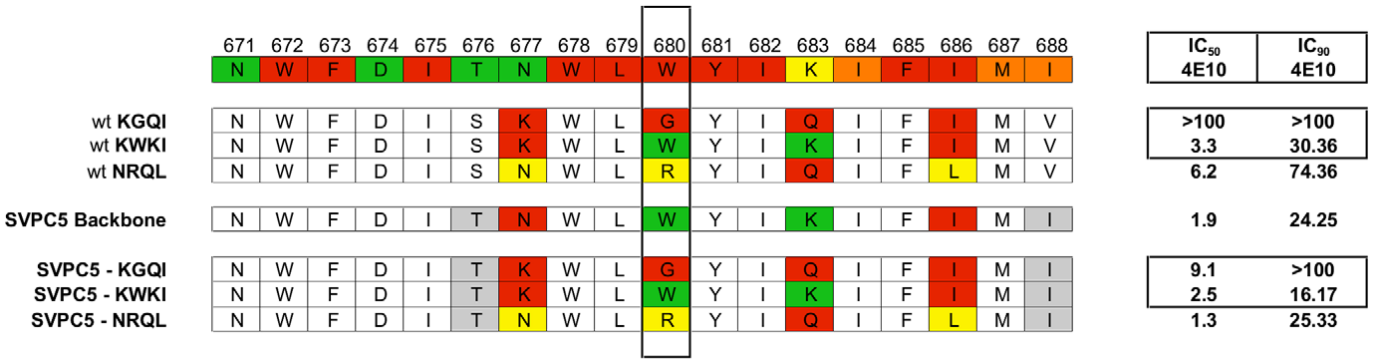
Comparison of wild-type and mutant MPER sequences. Alignment of representative subject sequences containing the 4-residue MPER cassettes used in mutagenesis study aligned against the same subtype C consensus used in previous tables. The wild-type SVPC5 MPER sequence and the sequence of all three SVPC5-MPER mutants are also aligned. Residues 677, 680, 683, and 686 have been arbitrarily colored for easy identification. Residues in grey represent differences between the SVPC5 backbone and the envs described in the study. IC_50_ and IC_90_ values for mAb 4E10 are displayed for each Env.

We next sought to determine if 4E10-resistance could be conferred on an unrelated 4E10-sensitive subtype C reference *env* (SVPC5) by substitution of the four amino acid MPER cassettes we identified from subject-pair 16 (residues 677, 680, 683, and 686) (Figure 4). The K_677_W_680_K_683_I_686_ (“KWKI”) and N_677_R_680_Q_683_L_686_ (“NRQL”) cassettes did not alter 4E10 sensitivity. The K_677_G_680_Q_683_I_686_ (“KGQI”) cassette reduced sensitivity to 4E10 by ∼5-fold. These data suggest that while position 680 is necessary for conferring the 4E10-resistant phenotype, additional changes outside the MPER are also required. The variations in 4E10 susceptibility in subject 12M's Env containing W680R are consistent with a model in which residues outside the MPER are critical for high-level resistance mediated by position 680.

Previously published *in vitro* mutagenesis data showed that a W680A substitution resulted in a marginal increase in 4E10 resistance at the IC_50_ level, but greatly increased resistance at the IC_90_ [22]. Since the 4E10-resistant Envs from subject-pair 16 also contained a small hydrophobic substitution (G), we examined the SVPC5-KGQI mutant for a similar phenotype. We found that it had an IC_90_ of >100 µg/mL while the R and W mutants had much lower IC_90_s (25.3 µg/mL and 16.17 µg/mL respectively). We also noted that the difference between IC_50_ and IC_90_ for the KWKI cassette is approximately 6-fold, while the difference between IC_50_ and IC_90_ for both the KGQI and NRQL cassettes is at least 10–20-fold (Figure 4).

## Discussion

*In vivo* neutralization escape and naturally occurring polymorphisms in the MPER region targeted by 3 of the most broadly neutralizing mAbs (4E10, 2F5 and Z13e1) are extremely rare [19], [20], [24], [25]. Moreover, a detailed structure of the complete functional envelope trimer is unknown [32], [54], [55], [56], [57], thus characterization of rare natural variants is an important source of relevant information on envelope structure/function.

In this study, we examined four subjects with naturally occurring MPER polymorphisms that confer resistance to mAb 4E10. In three cases, we showed clear evidence that resistance to 4E10 was driven by mutations in the extended epitope sequence (position 680), but that residues outside the MPER region were also essential for conferring the resistant phenotype. Our fourth case was similar to an *env* variant previously described [26], from a child with an anti-MPER neutralizing antibody response. However, in our subject we observed no changes in the LLP-2 region of *envs* [26].

When plasma and breast milk samples from subject 16M were tested for anti-MPER neutralization activity using an HIV-2/HIV-1 MPER chimera, none was observed. A simple binding ELISA showed modest reactivity of plasma from 16M against a MPER peptide, although relatively high binding to controls slightly weakens these data. We did not observe enhancement of neutralization titer with this plasma when assayed on cells expressing FcγRI, as had been observed previously for both mAbs 4E10 and 2F5 as well as for plasma containing MPER-reactive antibodies [51]. There are several possible explanations for these data. First is the possibility that MPER mutations in this subject are not driven by an antibody response, and that a significant anti-MPER activity is not present. Second, it is possible that anti-MPER antibodies target a complex epitope that includes position 680 but depends on other residues that differ from the HIV-2/HIV-1 subtype C consensus MPER chimera (residue 676 and/or 683). Third, anti-MPER activity may be present, but dependent on additional factors (e.g. complement, ADCC) not present in our *in vitro* assay systems. We currently favor a model wherein the targeted epitope is complex and not presented on the chimeric Env, but is weakly bound on the free peptide. Unfortunately, we have a very small volume of plasma available, which precludes the fine epitope mapping and binding-competition experiments required to resolve these questions.

It is of particular interest that in all cases in which 4E10 resistance was associated with a substitution at position 680, highly conserved substitutions elsewhere in the MPER were also present. Substitutions at position 680 were associated with changes at positions 677 and 683 in both of our subject-pairs, who were epidemiologically unlinked. While the patterns of substitution were different for each subject (and likely dependent on *env* context), our data suggest a cooperative role for positions 677, 680, and 683 in some important aspect of MPER function. This hypothesis is strengthened by the following observations: (1) that either 1 or 2 basic charges were conserved across this region of the MPER in *envs* from both subjects, (2) that the charge at position 683 is >99.9% conserved in subtype C, and (3) that this charge was absent in our Envs with substitutions at position 680 (which is itself >99% conserved). Recent structural studies place these amino acids as three of the four surface-exposed residues in the second (C-terminal) amphipathic helix of a helix-hinge-helix model of the MPER interacting with the viral membrane [21], [23]. Our data are consistent with this model, in that hydrophilic or charged substitutions observed in our Envs occurred at positions identified as surface-exposed in those studies. Furthermore, changes in 3 of the 4 surface-exposed residues comprising the second helix (arising as a consequence of mutations at position 680) suggest the preservation of an important interaction with either a distal part of Env or a component of the membrane itself that is required for full fusion activity.

Our findings also have implications for the mechanism of 4E10 binding. Specifically, in a recently proposed model [21], [23], 4E10-binding to gp41 occurs in a distinct, two-stage docking process, where the initial critical contact point for the mAb paratope is position 680, followed by alterations in the MPER secondary structure. These alterations eventually result in exposure of residues 672 and 673, which are then bound by the mAb in the second stage of the docking process. Our data are consistent with this model of binding, in that substitution of a small, hydrophobic residue at position 680 (G) had much greater effects on 4E10 binding at both the IC_50_ and IC_90_ levels than the substitution of a large, albeit basically charged, residue (R) at the same position. In fact, our substitution of a W680G containing cassette (KGQI) into an unrelated subtype C reference *env* produced neutralization data similar to that reported with the substitution of another small hydrophobic residue (A) for the tryptophan at position 680 [22]. It has also been recently shown that the fusion inhibitor 5-Helix, which binds to the C heptad repeat region of gp41 immediately N-terminal to the MPER, is K_on_ rate-restricted in it's neutralizing activity, such that K_on_ is a more dominant factor then equilibrium binding affinity [58]. We speculate that perhaps kinetic differences in MPER exposure between our resistant isolates and the SVPC5 reference Env may be responsible for the different effects of the described MPER substitutions on neutralization sensitivity.

These 680 mutations may also play a role in mediating sensitivity to T20, which binds to a fusion intermediate in gp41. While we observed a trend which did not reach statistical significance, a qualitative comparison of data from our primary isolates, and a JR2 mutant previously described, suggests a possible role for position 680 in mediating exposure of the heptad-repeat regions during the fusion process. This could come about through changes in tertiary/quaternary structure, as well as alterations in fusion kinetics. More detailed structural/functional studies will be necessary to test this hypothesis.

Several different models of the gp41 MSD have been proposed: the ‘classic’ model with a 25 residue MSD (683–707 HXB2 numbering) and a ‘snorkeling’ model with a 12 residue core between 683–696 (HXB2 numbering) which exposes the charged side-chains of residues K683 and R696 to the polar head groups of the lipid bilayer [53], [59]. Many of our gp41s have an exceedingly rare neutral substitution (K683Q), tied to the identity of residue 680. It has also been suggested that residues 679 to 683 of gp41 represent a cholesterol recognition/interaction amino acid consensus (CRAC) motif and that mutagenesis of this motif affects fusogenicity in a manner primarily dependent on the ability of the mutant Envs to bind cholesterol [60], [61]. In a recent study of *in vitro* generated MPER mutants, a W680G substitution was found to have the smallest negative impact on Env fusogenicity [60]. This is one possible explanation for why the W680G mutation was favored in subject 16M at the early time point and why it was transmitted. Data from this study also indicated that non-CRAC sequences, if they retained sufficient cholesterol binding activity, could also facilitate fusion [60]. Of the seven major MPER sequence variants we identified in subject-pairs 12 and 16, four of them had substitutions at position 683 that eliminated the CRAC motif (K683Q), yet were functional and represented significant portions of those subject's quasi-species. We did not examine these MPER peptides for cholesterol-binding activity, but such experiments would prove useful in further defining the functional requirements for this region of gp41. Taken together, these data all suggest a functionally critical interaction between the MPER and the MSD (likely including distal portions of Env) and potentially tied to stabilization of gp41 within the viral membrane during the fusion process.

In summary, we have described four subjects (three mothers and one of their infants) with HIV envelopes highly resistant to mAb 4E10 as a result of rare polymorphisms in the MPER (substitution frequencies of 0.07%–0.43% per site). The high frequency of MPER polymorphisms in our cohort is remarkable compared to other studies [19], [20], [24], [25]. It is interesting to speculate that this may be due to pregnancy-induced changes in B cell biology. Notably, the only other naturally 4E10 resistant variant was in a perinatally infected child [26] and envelopes from 3/10 pregnant Kenyan women were resistant to 4E10 but no genetic basis for this resistance was documented [27].

In one case, the virus was fit enough to transmit, indicating that these mutations are not associated with an insurmountable fitness cost. However, the dominant W680G variant in this subject almost completely disappeared three years later, suggesting that whatever pressure selected for this variant was transitory in nature. We have also shown that the resistant phenotype seen with position 680 mutations requires the participation of residues outside the MPER, as well as compensatory changes within the MPER itself. These findings have important implications for vaccine targeting of the MPER region. Additionally, these isolates provide useful tools for probing the structure-function relationship of the envelope protein.
